# A 10-week play-based after-school program to improve coordinative abilities and physical fitness capabilities among adolescents: a randomized trial

**DOI:** 10.1038/s41598-024-61275-0

**Published:** 2024-06-12

**Authors:** M. Kurnaz, F. Flôres, M. Altınkök, H. T. Esen, A. F. Silva

**Affiliations:** 1 Faculty of Sport Sciences, Department of Physical Education and Sport Teaching, Haliç University, 34060 Istanbul, Turkey; 2 Piaget Research Center for Ecological Human Development, Instituto Piaget, Lisbon, Portugal; 3 Research Center in Sports Performance, Recreation, Innovation and Technology (SPRINT), 4960-320 Melgaço, Portugal; 4 Faculty of Sport Sciences, Department of Physical Education and Sports, Akdeniz University, 07058 Antalya, Turkey; 5 Escola Superior Desporto e Lazer, Instituto Politécnico de Viana do Castelo, Rua Escola Industrial e Comercial de Nun’Álvares, 4900-347 Viana do Castelo, Portugal

**Keywords:** Sports didactics, Physical education and sport, Physical fitness, Team sports, Adolescents, Physiology, Health care

## Abstract

The average levels of physical activity in children are below the ideal, which may influence children's health and motor competence levels. Therefore, this study aimed to assess the impact of a 10-week play-based after-school program on 50 twelve-year-old students’ anthropometric characteristics (body height and body weight), coordinative abilities (flamingo balance test and T-test agility), and physical fitness (sit and reach, 20-m sprint test, SLJ, CMJ, and handgrip). After baseline assessments, the students were randomly divided into two groups: one participating in a play-based after-school program (experimental group) and the other attending regular PE classes (control group). No differences were noted between the sexes. Analysis revealed baseline differences between groups in body weight, agility T-test, and right-handgrip, with no significant sex differences. Following the intervention, the experimental group demonstrated improvements in the 20-m sprint test (F(1,46) = 11.03, p < 0.01), flamingo balance test (F(1,46) = 9.16, p = 0.004), SLJ (F(1,46) = 5.30, p = 0.03), agility T-test (F(1,46) = 28.30, p < 0.01), and right-handgrip (F(1,46) = 6.59, p < 0.01). In summary, the results suggested that a 10-week play-based after-school program enhances coordinative abilities and physical fitness in 12-year-old children. This underscores the potential advantages of integrating play-based physical activities into schools to promote holistic health and fitness in children.

## Introduction

The global prevalence of physical inactivity and obesity has escalated in recent years^1^. These alarming developments underscore the urgent need to disseminate information and evaluate the importance of physical activity (PA) in our society^2,3^. Research has consistently demonstrated that increased levels of PA are associated with health benefits, including decreased body fat, heightened cardiorespiratory fitness, augmented muscular strength, enhanced bone health, and cognitive and psychosocial advantages^1,4,5^. Remarkably, these advantages apply to individuals of all age groups, from youth to adults^3,6^. On the other hand, inadequate levels of PA pose a potential threat to the next generation of active people. To clarify, reducing the “minimum thresholds” (or at the very least, the percentage of young individuals capable of meeting these thresholds) for physical capabilities and motor skill performance would curtail the potential of future athletes in terms of both quantity and quality. Therefore, numerous studies have consistently demonstrated a substantial connection between sports participation and increased PA^7^, motor competence^8,9^, and overall fitness levels^10,11^.

The engagement of young individuals in PA is known to be influenced by a complex array of psychological, biological, social, cultural, and environmental factors, which have the potential to impact their decisions regarding the adoption and sustained commitment to a physically active lifestyle^12^. Indeed, the concept of physical literacy encompasses these aspects, arguing that a rich motor experience, especially at young ages, will promote personal fulfillment, self-confidence, and self-esteem throughout life^13^. Hence, it is crucial to explore the strategies that can be implemented to mitigate the decline in PA levels among youth, while fostering physical literacy and its associated benefits. One noteworthy aspect is the motivation to engage in PA, with a particular focus on the role of physical education (PE) classes as potential influencers. Even a few decades ago, Sallis and McKenzie^14^ contended that fostering positive motivation among students within the PE context could potentially shape children's proclivity toward adopting physically active lifestyles in adulthood. Subsequently, Harter’s competence motivation theory^15,16^ proposed deriving enjoyment from PA is a consequence of experiencing success and mastery, which, in turn, bolster an individual's perception of their competence. This perspective is substantiated by several empirical studies^17–19^ which consistently demonstrate a significant positive association between perceived competence and participation in PA. This perspective is also in line with the concept of Physical Literacy^20^, which focuses on the development of motivation, self-assurance, physical proficiency, knowledge, and understanding, essential for the lifelong maintenance of PA^13,20^.

A physically literate individual embodies a skilled mover with an extensive repertoire of well-practiced movement responses honed through exposure to a diverse array of challenging environments. Furthermore, this individual demonstrates keen perceptiveness in interpreting every facet of the physical surroundings, anticipating movement requirements or opportunities, and responding with intelligence and creativity^13,20^. Considering that effective motor skill execution hinges on an efficient combination of cognitive processing, the mastery of fundamental movement patterns, and the proficient generation and absorption of force^21^, attaining physical literacy is best achieved through a rich learning context, such as play-based activities, rather than isolated skill acquisition^22^. Furthermore, the literature has presented numerous studies that have shown the effectiveness of various play-based after-school programs^23–26^. Thus, it's essential to acknowledge that any form of movement, whether it involves jumping, bracing, lifting, catching, accelerating, or decelerating, necessitates the generation or absorption of force to some degree. Consequently, the enhancement of muscle and connective tissue strength should be regarded as a vital complement to the development of coordination and overall motor skill proficiency^27^. This perspective has resulted in observed improvements in most fitness test outcomes and motor competence variables both at the initial assessment and during follow-up among those who engaged in multiple sports^28^.

Considering the above mentioned and as far as we know, the present study aimed to understand the effects of a 10-week play-based after-school intervention on children’s anthropometric characteristics (body height and body weight), coordinative abilities (equilibrium and agility), and physical fitness (flexibility, 20-m sprint test, and strength). It was hypothesized that a supplementary 40-min (twice-a-week) intervention would be enough to improve the anthropometric characteristics, coordinative abilities, and physical fitness compared to the control group.

## Materials and methods

### Participants

This research constituted a single-blind controlled randomized experimental trial aimed at comparing the efficacy of a play-based after-school program against a standard physical education program. The study involved fifty 12-year-old students from a singular educational institution who were recruited for this investigation. The inclusion criteria included: (i) have the target age; (ii) not having reported any injuries in the last month; (iii) having the informed consent signed by the legal guardian; iv) having participated in at least 80% of the training sessions. As exclusion criteria, the following were considered: (i) being above or below the age range of 11 and 13 years, respectively; (ii) reporting any injury or muscular and/or joint discomfort; (iii) not having the informed consent signed by the legal guardian; and (iv) missing the participation of more than 20% of the training sessions. A priori sample size calculation was performed using a free online tool, G*Power1, with a power level of 80% and an α level of 0.05 and revealed that the sample size of 46 would be sufficient for the analysis. Upon conducting baseline assessments, these students were blinded randomly assigned to two distinct groups: the experimental group, which participated in the play-based after-school program, and the control group, which exclusively attended standard physical education classes. Using numbers attributed to each subject the randomization was carried out through a research randomizer (https://www.randomizer.org/), allocating each individual in a group (experimental or control groups). Throughout the intervention period, participants were instructed to abstain from involvement in any structured sports activities. Before the initiation of the intervention, both participants and their respective parents or guardians were duly informed about the experimental procedures and provided informed consent by signing an appropriate form. The Ethics Committee of Piaget Institute (IPEC) (Protocol ID: P02-S09-27.04.2022; Date: 27.04.2022) and Haliç University Non-Interventional Research Ethics Committee (Protocol ID: 239; Date: 31.10.2023) approved all protocols employed in this study. The principles outlined in the Declaration of Helsinki were meticulously adhered to throughout the entirety of this research endeavor.

### Procedures

**Figure 1:**
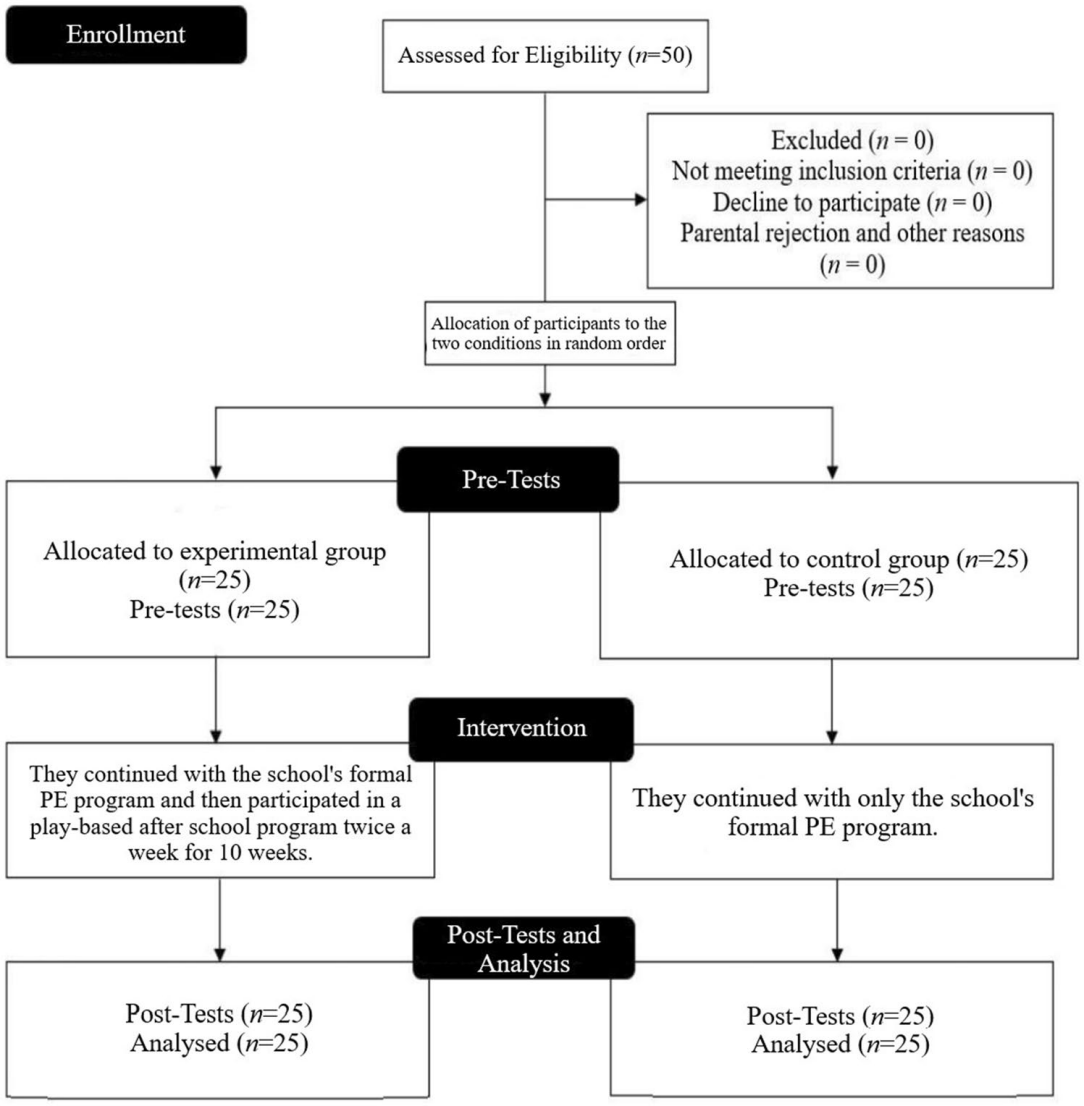
Flow chart.

Both groups engaged in standard physical education sessions twice weekly for 40 min, while the experimental group additionally took part in a play-based after-school program (Fig. 1). The play-based sessions occurred twice a week after regular school hours, spanning 40 min each, over 10 weeks. Each session commenced with a standardized 5-min warm-up involving moderate-intensity running and exercises relevant to the specific game or sport scheduled for that session. The primary segment of the session comprised two distinct games, each played for 30 min. Post the main activity, a 5-min cooldown exercise was implemented. The program predominantly featured small-sided games encompassing football, basketball, handball, and volleyball, known for their high engagement and training intensity suitable for all children^29^. For logistical purposes, varying small games were occasionally played, altering player numbers and field sizes, and both outdoor and indoor facilities were utilized based on weather conditions. In contrast, the control group exclusively participated in standard physical education activities outlined for that academic term, focusing on training across different team and individual sports.

The play-based after-school program’s duration was chosen following suggestions from earlier investigations. According to the majority of research, the program typically lasts between 10 and 12 weeks, with sessions lasting one to two hours each week^26,30–32^. It has been shown that a length of 10–12 weeks is optimal for attaining significant outcomes in the enhancement of coordination skills and physical fitness levels. This length of time strikes a compromise between offering participants a practically feasible intervention and making it sufficiently intense and is in line with established practices in the field. Activities for adolescents aged 12 were incorporated into the play-based after-school program. Developmental considerations served as the foundation for the program’s selection of 12-year-old participants. This age range is crucial for the development of coordinative abilities and physical fitness capabilities.

### Data collection tools

#### Anthropometric characteristics

Anthropometric characteristics were collected, with body weight being measured using a calibrated beam scale with a precision of 0.1 kg, and the body height determined to the nearest 0.5 cm utilizing a stadiometer affixed to the wall. The body weight was measured at the beginning and end of the intervention, but height was only assessed at the beginning.

#### Countermovement jump (CMJ)

The vertical jump performance was evaluated using a Countermovement jump test. The starting position was stationary and upright, with the knees completely extended and the hands maintained at the waist. Subjects squatted to their desired height before initiating a strong ascending motion. Subjects were instructed to avoid bending hips, knees, and ankles during flight and touchdown to minimize the impact on jump height calculations based on flight time phase duration. Finally, they were encouraged to leap as high as possible, with verbal encouragement appied during the jumps. Subjects made three tries with a passive recovery time of 45 s between leaps, and the best result was recorded for data analysis. The jump height (cm) was measured using the New Test 2000 performance analysis system [Newtest Oy in Oulu, Finland]^33,34^.

#### Standing long jump (SLJ)

The evaluation of explosive leg power was conducted through the administration of the SLJ test. Participants received instructions to maintain an erect posture, execute a forceful upward thrust with both feet on the ground, and propel themselves forward while aiming for maximal height during the jump. This movement involved initiating the jump with a two-foot takeoff and incorporating arm swinging during the landing phase. A standard measuring tape was used to gauge the distance between the take-off line and the closest point where the back of the heel made contact upon landing. Following two attempts and a passive recovery period of 60 s between jumps, the most favorable outcome in centimeters was documented and utilized for subsequent data analysis^33,35^.

#### Flamingo balance (static) test

The assessment of static balance utilized the Flamingo Balance Test, evaluating both the dominant and non-dominant extremities of the child. Participants were requested to remove their shoes before the test commenced. Initially, participants were instructed to place their dominant extremity onto a beam, followed by bending the knee of their contralateral leg and gripping the ankle of the contralateral leg (opposite to their dominant extremity) using one hand. The duration for which participants maintained these positions was recorded, noting the duration they sustained the posture without losing balance. Within 1 min, the total count of falls was tallied, constituting the static balance score. If a child experienced 15 or more falls within 30 s, the test was halted, and a score of zero was documented as an indication of failure^36–38^.

#### Sit-and-reach test

The evaluation of participants’ flexibility in the hips and waist involved conducting a sit-and-reach test. Participants were directed to extend their knees fully and position their ankles on the floor in neutral dorsiflexion against the sit-and-reach box. With hands placed on the box's surface, participants were instructed to gradually extend their knees forward, endeavoring to reach as far forward as possible along the top surface of the box. The measurement was taken in centimeters from the surface to the point where the middle fingertips landed^39^. Each participant performed the test three times, with the highest achieved result being considered for assessment purposes.

#### 20-m sprint test

The assessment of participants' sprint performance in the study involved the utilization of the 20-m sprint test. Participants positioned themselves with their feet on the starting line, and upon the signal of a whistle, were instructed to cover the 20-m distance between marked starting and finishing points as swiftly as possible. The time taken to complete the sprint was accurately measured by a designated timekeeper. This test was administered in duplicate, allowing participants a period of rest between trials. The recorded performance considered for analysis was the shortest duration achieved among the two test trials, documented in seconds^37,40,41^.

#### Hand grip strength

The evaluation of hand grip strength involved the use of a digital hand dynamometer [T.K.K. 5401 Grip-D; Takey, Tokyo, Japan], with the results documented in kilograms. The dynamometer boasted a reported precision of 0.1 kg, ensuring accurate measurement of participants’ hand grip strength. The subjects were asked to keep the shoulder in adduction and neutral rotation, the elbow in 90 degrees of flexion, and the forearm and wrist in a neutral position. Subjects were then asked to squeeze as hard as possible without grasping the width with their dominant hand for 5 s while the tool rested comfortably in their hand. Each test was repeated three times with a 30-s break in between and the best result was recorded^42^.

#### Agility T-test

This test serves as an agility assessment encompassing forward, lateral, and backward running, gauging both defensive maneuvers and speed with directional changes. Each participant engaged in a sequence involving a 9-m sprint forward, touching the tip of a cone with their right hand. Subsequently, they shuffled 4.5 m to the left and touched the apex of another cone with their left hand. Altering direction, participants then covered a 9-m distance to the right, placing their right palm on the tip of a separate cone. Next, they moved 4.5 m to the left, reaching out with their right hand to touch the center of a cone's tip. Concluding the trial, participants backtracked 9 m, crossing the finish line^43^. The recorded time for the quickest performance out of the two trials was documented for analysis.

### Statistical analysis

The descriptive statistics are presented in the form of mean and standard deviation. The normality of the data was used using the Shapiro–Wilk test. An independent sample t-test was conducted to analyze if differences existed between the experimental and the control group in all independent variables (independently of sex). A mixed-ANOVA for repeated measures was used to compare height, weight, and the results on the flamingo balance test, sit and reach, 20-m sprint test, agility T-test, and handgrip regarding their sex and group (control or experimental group). When interactions were noticed, a paired sample t-test was conducted, analyzing pre- and post-evaluations in each group (independently of sex). The Statistical Package for Social Sciences [SPSS, v. 29.0] software was used, with an alpha significance level of 0.05.

## Results

Out of the 50 participants, 25 were included in the experimental group and the remaining in the control group. All of them completed the intervention, thus all were eligible for analysis.

**Figure 2:**
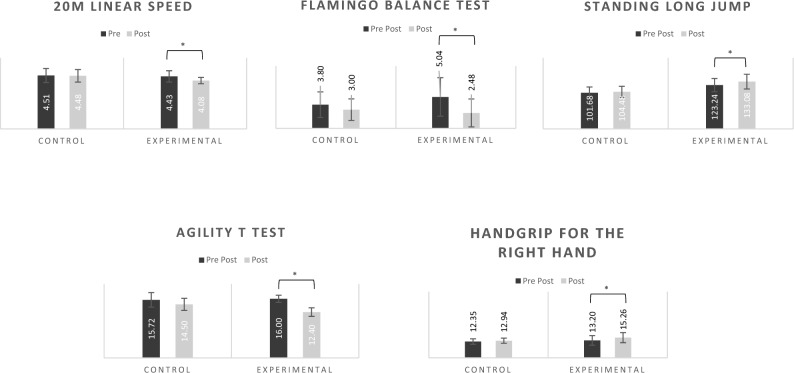
Pre and Post results in the control and experimental groups regarding the tests of 20 m linear speed, flamingo balance, standing long jump, agility t-test and handgrip. The * means statistically significant differences.

Differences were noticed between groups in the baseline moment (pre-test) on body weight (F(1,48) = 5.05, p = 0.03), on the agility T-test (F(1,48) = 13.21, p < 0.01), and handgrip for the right hand (F(1,48) = 7.43, p = 0.01) (See Table 1, for more information). No difference was noted between the sexes. When comparing the two evaluation moments (pre and post) interactions between the groups and the moments were observed in the following tests: 20-m sprint test (F(1,46) = 11.03, p < 0.002), Flamingo balance (F(1,46) = 9.16, p < 0.01), SLJ (F(1,46) = 5.30, p = 0.03), agility T-test (F(1,46) = 28.30, p < 0.01) and handgrip for the right hand (F(1,46) = 6.59, p < 0.01). Therefore, in those variables, an analysis between moments (pre and post) was conducted showing differences in all of them: 20-m sprint test (t(1,49) = 3.98, p < 0.01), flamingo balance test (t(1,49) = 5.23, p < 0.01), SLJ (t(1,49) = 33.01, p < 0.01), agility T-test (t(1,49) = 9.35, p < 0.01) and handgrip for the right hand (t(1,49) = − 4.80, p < 0.01). Figure 2 shows the comparison between pre and post on those variables.

**Table 1:** Mean and standard deviation for males, females, and both (total) for body mass, body weight, 20 m linear speed test, Flamingo balance test, Standing Long Jump, Counter Movement Jump, sit and reach, agility T test, right and left handgrip.

	Control group						Experimental group					
	Males (n = 7)		Females (n = 18)		Total (n = 25)		Males (n = 17)		Females (n = 8)		Total (n = 25)	
	Pre	Post	Pre	Post	Pre	Post	Pre	Post	Pre	Post	Pre	Post
Body mass (kg)^a^	41.58 ± 7.16	42.86 ± 7.45	40.90 ± 12.07	42.72 ± 12.73	41.09 ± 10.78	42.76 ± 11.35	39.82 ± 7.85	41.13 ± 8.46	35.87 ± 4.89	36.64 ± 4.95	38.56 ± 7.19	39.69 ± 7.71
20 m linear speed test (s)^b^	4.18 ± 0.48	4.14 ± 0.50	4.63 ± 0.58	4.61 ± 0.50	4.51 ± 0.59	4.48 ± 0.53	4.40 ± 0.43	4.03 ± 0.26	4.49 ± 0.60	4.17 ± 0.30	4.43 ± 0.48	4.08 ± 0.27
Flamingo balance (s)^b^	3.29 ± 1.98	3.29 ± 1.80	4.00 ± 2.11	2.89 ± 1.75	3.80 ± 2.06	3.00 ± 1.73	5.71 ± 3.22	3.12 ± 2.26	3.62 ± 2.39	1.13 ± 1.55	5.04 ± 3.09	2.48 ± 2.24
Standing long jump (cm)^b^	102.71 ± 22.24	107.00 ± 20.72	101.28 ± 14.00	103.50 ± 134.29	101.68 ± 16.25	104.48 ± 15.29	123.88 ± 17.75	134.29 ± 23.12	121.88 ± 21.71	130.50 ± 15.83	123.24 ± 18.66	133.08 ± 20.80
Counter movement jump (cm)	22.29 ± 5.31	25.43 ± 4.47	19.17 ± 3.09	21.67 ± 2.74	20.04 ± 3.98	22.72 ± 3.65	21.94 ± 3.01	23.82 ± 3.30	21.63 ± 4.31	23.88 ± 4.39	21.84 ± 3.39	23.84 ± 3.59
Sit and reach (cm)	17.71 ± 3.45	20.86 ± 2.55	19.06 ± 2.51	21.44 ± 1.65	18.68 ± 2.80	21.28 ± 1.90	21.59 ± 3.45	24.71 ± 4.04	20.63 ± 1.77	25.00 ± 3.16	21.28 ± 3.01	24.80 ± 3.72
Agility t-test (s)^a,b^	15.14 ± 2.67	13.52 ± 1.68	15.94 ± 1.66	14.88 ± 1.51	15.72 ± 1.97	14.50 ± 1.64	15.88 ± 0.78	12.35 ± 1.22	16.25 ± 1.28	12.50 ± 1.07	16.00 ± 0.96	12.40 ± 1.16
Handgrip right (N)^a,b^	13.19 ± 1.67	13.31 ± 1.39	12.03 ± 2.09	12.80 ± 2.14	12.35 ± 2.02	12.94 ± 1.94	13.71 ± 4.12	16.06 ± 4.11	12.11 ± 1.99	13.55 ± 2.38	13.20 ± 3.61	15.26 ± 3.79
Handgrip left (N)	12.10 ± 1.30	14.941.46 ±	12.08 ± 2.77	13.75 ± 2.05	12.09 ± 2.42	13.75 ± 2.02	12.84 ± 3.68	14.28 ± 4.21	11.13 ± 1.76	13.88 ± 2.79	12.29 ± 3.25	14.15 ± 3.76

^a^Differences were noticed between groups in the baseline moment (pre-test); ^b^Time × group interaction observed.

## Discussion

The present investigation examined the effects of a 10-week play-based after-school intervention on anthropometric characteristics, coordinative abilities, and physical fitness among 12-year-old students. It is important to note that our findings revealed that there may have been inherent differences between the two groups before the intervention, particularly concerning variables such as body weight, performance on the agility T-test, and right-hand grip strength. Notably, the highest levels of these variables were found in the experimental group. This aligns with the literature, which also documented that children constitute a distinct population with a wide range of anthropometric and physical characteristics^44,45^. However, the distribution of subjects by sex across the groups may have exerted some influence on the current study.

Unsurprisingly, in the experimental groups, the flamingo balance test, the SLJ, agility T-test, and the handgrip for the right-hand improved performance from the baseline to the 10^th^ week. Therefore, this observation is consistent with those of previous investigations conducted on school-aged children^46,47^. Moreover, numerous investigations have underscored the effectiveness of various play-based after-school programs^23,25,26,30,48^. Gao and colleagues^23^, focused on evaluating the impact of a school-based exergaming intervention on children’s perceived competence, motor competence, and physical activity. Their results indicated positive effects of exergaming in promoting moderate-to-vigorous PA among children, thereby enhancing their perceived competence and motor competence. In a study conducted by Raju and colleagues^48^, the impact of an after-school intervention program on health-related physical fitness components in secondary school boys was investigated. The study's results showed that the experimental group, which participated in a 10-week football training intervention, exhibited enhanced cardiovascular endurance and flexibility among the participants. Hence, effective motor skill execution requires a combination of cognitive processing and mastery of fundamental movement skills. Therefore, physical literacy can be achieved through a rich learning context, such as play-based activities, rather than isolated skill acquisition.

Interventions applied in Physical Education classes using the HIIT methodology have shown that other approaches may also influence cardiorespiratory capacity, including parameters related to blood pressure, as well as body composition^4,49,50^. In a systematic review aiming to understand the effect of implementing the HIIT methodology to improve body composition and cardiorespiratory capacity in obese children, it was observed that 2 to 3 times per week this methodology could be a viable strategy for enhancing children's body composition and health^4,49,50^. Thus, our findings were allied to these previous investigations, demonstrating significant improvements across multiple fitness components from the start of the intervention (pre-test) to the end of the 10 weeks (post-test).

It's worth delving into the discrepancy observed in the CMJ test results, which deviate from findings in previous studies involving a similar population^51,52^. According to the literature, the CMJ is different regarding sex, with boys demonstrating greater jumping height through applying a larger concentric impulse and, thus, achieving greater velocity throughout most of the concentric phase, including the take-off^53,54^. In addition, Focke et al.^53^ suggest that age, sex, and activity level of children and adolescents affect jumping performance and its variability. Consequently, these aspects must be considered when evaluating this population.

It is important to point out that the increases were found in the 20-m sprint test, the flamingo balance test, the SLJ, the agility T-test, and the handgrip strength for the right-hand tests. Hence, our results suggest that a 10-week play-based after-school program had a positive impact on children's physical fitness and coordinative abilities throughout the intervention, showing that the design of polythematic team-sports sessions can improve children’s physical fitness and coordinative abilities. Lourenço and colleagues^55^ also found similar results using polythematic classes. The authors suggest that PE classes using more the one team sport exercise can improve children's PA levels. Several reasons may account for the results found in the present investigation: first, children may have improved as they age; second, the test scores improved partially owing to a learned effect at the follow-up assessments; and finally, the simple fact of improved children the number of hours in exercises (10-week intervention) may have improved owing to physical activity participation.

The general positive outcomes observed in our results align with previous research that emphasizes the benefits of play-based physical activities for children's development^24–26,48^. Engaging in polythematic exercises during an extracurricular activity not only contributes to physical fitness but also enhances the coordination, balance, and agility of the students. These findings emphasize the importance of incorporating such team sports play-based interventions into school curricula to promote overall health and well-being among 12-year-old children.

## Limitations and future directions

The existing literature highlights the challenges of designing play-based sports interventions for children due to their varying levels of development, learning abilities, and competence^56,57^. Hence, it is also critical to acknowledge certain limitations of the present investigation. For instance, the distribution between groups was not balanced regarding sex, and sex differences may have influenced our results, especially regarding CMJ as previously indicated in the literature^53,54^. The disparity in participant representation between boys and girls may introduce bias and affect the validity of sex-related conclusions drawn from the research. This imbalance may not accurately reflect the motor performance of all sex groups, potentially limiting the generalizability of the findings. Future research should aim for a more balanced representation of sexes to ensure more comprehensive and accurate conclusions regarding sex-related phenomena. Finally, the additional 40 min of physical exercise provided to the experimental group can influence the results and need to be more investigated, compared to other forms of intervention.

Acknowledging the limitations of our study, it's critical to address potential solutions or recommendations for future research. Thus, further investigations could explore the long-term effects of such interventions and delve into potential gender differences in response to the sports program. Furthermore, considering the duration and intensity of the intervention, it may be valuable to assess whether these improvements are sustained over time or require ongoing participation. Lastly, evaluating participants’ levels of motor competence can also be important, due to the strong relationship of this variable to physical levels^17,58^. To gain a more comprehensive understanding of the intervention's long-term effects, forthcoming research might encompass follow-up evaluations conducted several months or possibly years following the intervention. This extended duration would enable us to evaluate the durability of the observed enhancements over time and contemplate a wider implementation to enhance the overall welfare of school-aged children.

## Conclusion

To summarize, it seems that this play-based after-school intervention could be considered to increase PA among 12-year-old children and improve coordination skills and physical fitness. Although differences between groups were observed before the intervention in body weight, performance on the agility T-test, and right-hand grip strength, after the intervention, these differences persisted and the 20-m sprint test, Flamingo balance, and SLJ tests also stood out in the experimental group. Therefore, the present study highlights the potential benefits of integrating play-based physical activities into the school environment, emphasizing the importance of promoting holistic health and fitness in children.

## Data Availability

The datasets generated during and analyzed during the current study are available from the corresponding author upon reasonable request.

## References

[1] Ayala-Marín AM, Iguacel I, Miguel-Etayo PD, Moreno LA (2020). Consideration of social disadvantages for understanding and preventing obesity in children. Front. Public Health.

[2] Bull FC, Al-Ansari SS, Biddle S, Borodulin K, Buman MP, Cardon G, Carty C, Chaput JP, Chastin S, Chou R (2020). World Health Organization 2020 guidelines on physical activity and sedentary behaviour. Br. J. Sports Med..

[3] Piercy KL, Troiano RP, Ballard RM (2018). The physical activity guidelines for americans. JAMA.

[4] Alvarez C, Ramírez-Campillo R, Ramírez-Vélez R, Izquierdo M (2017). Effects of 6-weeks high-intensity interval training in schoolchildren with insulin resistance: Influence of biological maturation on metabolic, body composition, cardiovascular and performance non-responses. Front. Physiol..

[5] Domaradzki J, Koźlenia D, Popowczak M (2022). Prevalence of positive effects on body fat percentage, cardiovascular parameters, and cardiorespiratory fitness after 10-week high intensity interval training in adolescents. Biology.

[6] An HY, Chen W, Wang CW, Yang HF, Huang WT, Fan SY (2020). The relationships between physical activity and life satisfaction and happiness among young, middle-aged, and older adults. Int. J. Environ. Res. Public Health.

[7] Wickel EE, Eisenmann JC (2007). Contribution of youth sport to total daily physical activity among 6- to 12-yr-old boys. Med. Sci. Sports Exerc..

[8] da Roche Queiroz D, Ré AHN, dos Santos Henrique R, de Sousa Moura M, Cattuzzo MT (2014). Participation in sports practice and motor competence in preschoolers. Motriz Rev. Educ. Fís..

[9] Toivo K, Vähä-Ypyä H, Kannus P, Tokola K, Alanko L, Heinonen OJ, Korpelainen R, Parkkari J, Savonen K, Selänne H, Kokko S, Kujala UM, Villberg J, Vasankari T (2023). Physical activity measured by accelerometry among adolescents participating in sports clubs and non-participating peers. Eur. J. Sport Sci..

[10] Fransen K, Vanbeselaere N, De Cuyper B, Coffee P, Slater MJ, Boen F (2014). The impact of athlete leaders on team members’ team outcome confidence: A test of mediation by team identification and collective efficacy. Sport Psychol..

[11] Hardy LL, Reinten-Reynolds T, Espinel P, Zask A, Okely AD (2012). Prevalence and correlates of low fundamental movement skill competency in children. Pediatrics.

[12] Laddu D, Paluch AE, LaMonte MJ (2021). The role of the built environment in promoting movement and physical activity across the lifespan: Implications for public health. Prog. Cardiovasc. Dis..

[13] Whitehead M (2010). Physical Literacy: Throughout the Lifecourse.

[14] Sallis JF, McKenzie TL (1991). Physical education’s role in public health. Res. Q. Exerc. Sport.

[15] Harter S, Roberts GC, Landers DM (1981). The development of competence motivation in the mastery of cognitive and physical skills: Is there still a place for joy?. Psychology of Motor Behavior and Sport.

[16] Klint KA, Weiss MR (1987). Perceived competence and motives for participating in youth sports: A test of harter’s competence motivation theory. J. Sport Psychol..

[17] Stodden D, Langendorfer S, Roberton MA (2009). The association between motor skill competence and physical fitness in young adults. Res. Q. Exerc. Sport.

[18] Timo J, Sami YP, Anthony W, Jarmo L (2016). Perceived physical competence towards physical activity, and motivation and enjoyment in physical education as longitudinal predictors of adolescents’ self-reported physical activity. J. Sci. Med. Sport.

[19] Tsuda E, Goodway JD, Famelia R, Brian A (2020). Relationship between fundamental motor skill competence, perceived physical competence and free-play physical activity in children. Res. Q. Exerc. Sport.

[20] Whitehead M (2001). The concept of physical literacy. Eur. J. Phys. Educ..

[21] Lloyd, R. S., Moeskops, S. & Granacher, U. Motor skill training in young athletes. in *Strength and Conditioning for Young Athletes*, 103–130 (2019).

[22] Pot N, van Hilvoorde I, Afonso J, Koekoek J, Almond L (2017). Meaningful movement behaviour involves more than the learning of fundamental movement skills. Int. Sports Stud..

[23] Gao Z, Zeng N, Pope ZC, Wang R, Yu F (2019). Effects of exergaming on motor skill competence, perceived competence, and physical activity in preschool children. J. Sport Health Sci..

[24] Gao Z, Wang R (2019). Children’s motor skill competence, physical activity, fitness, and health promotion. J. Sport Health Sci..

[25] Li H, Cheong JPG, Hussain B (2023). The effect of a 12-week physical functional training-based physical education intervention on students’ physical fitness: A quasi-experimental study. Int. J. Environ. Res. Public Health.

[26] Petrušič T, Trajković N, Bogataj Š (2022). Twelve-week game-based school intervention ımproves physical fitness in 12–14-year-old girls. Front. Public Health..

[27] Radnor JM, Moeskops S, Morris SJ, Mathews TA, Kumar NTA, Pullen BJ, Meyers RW, Pedley JS, Gould ZI, Oliver JL, Lloyd RS (2020). Developing athletic motor skill competencies in youth. Strength Cond. J..

[28] Salin K, Huhtiniemi M, Watt A, Mononen K, Jaakkola T (2021). Contrasts in fitness, motor competence and physical activity among children involved in single or multiple sports. Biomed. Hum. Kinet..

[29] Bendiksen M, Williams CA, Hornstrup T, Clausen H, Kloppenborg J, Shumikhin D (2014). Heart rate response and fitness effects of various types of physical education for 8- to 9-year-old school children. Eur. J. Sport Sci..

[30] Curry C, Knijnik JD, Chepyator-Thomson JR, Hsu SH (2013). Physical education and the after-school sports program in Australian schools: Barriers and challenges for the 21st century. Global Perspectives on Physical Education and After-School Sport Programs.

[31] Kolovelonis A, Pesce C, Goudas M (2022). The effects of a cognitively challenging physical activity intervention on school children’s executive functions and motivational regulations. Int. J. Environ. Res. Public Health.

[32] Larsen MN, Nielsen CM, Helge EW, Madsen M, Manniche V, Hansen L, Hansen PR, Bangsbo J, Krustrup P (2018). Positive effects on bone mineralisation and muscular fitness after 10 months of intense school-based physical training for children aged 8–10 years: The FIT FIRST randomised controlled trial. Br. J. Sports Med..

[33] Dello Iacono A, Holgado Lopez C, Bakhshi A, Halperin I (2022). The isometric horizontal push test correlates with jumping and sprinting performance among athletes and recreationally active controls. Biol. Sport.

[34] Enoksen E, Tønnessen E, Shalfawi S (2009). Validity and reliability of the newtest powertimer 300-series® testing system. J. Sports Sci..

[35] Porter JM, Ostrowski EJ, Nolan RP, Wu WFW (2010). Standing long-jump performance is enhanced when using an external focus of attention. J. Strength Cond. Res..

[36] Adam C, Klissouras V, Ravazzolo M, Renson R, Tuxworth W (1987). Eurofit: European Test of Physical Fitness.

[37] Mackenzie B (2005). 101 Performance Evaluation Test.

[38] Rodriguez FA, Valenzuela A, Gusi N, Nacher S, Gallardo I (1998). Evaluation of the health-related fitness in adults (II): Reliability, feasibility and reference norms by means of the AFISALINEFC. Apunts Educ. Fis. Dep..

[39] Cornbleet SL, Woolsey NB (1996). Assessment of hamstring muscle length in school-aged children using the sit-and-reach test and the ınclinometer measure of hip joint angle. Phys. Ther..

[40] Granacher U, Borde R (2017). Effects of sport-specific training during the early stages of long-term athlete development on physical fitness, body composition, cognitive, and academic performances. Front. Physiol..

[41] Roth A, Schmidt SCE, Seidel I, Woll A, Bös K (2018). Tracking of physical fitness of primary school children in trier: A 4-year longitudinal study. BioMed Res. Int..

[42] Roberts HC, Denison HJ, Martin HJ, Patel HP, Syddall H, Cooper C, Sayer AA (2011). A review of the measurement of grip strength in clinical and epidemiological studies: Towards a standardised approach. Age Ageing.

[43] Torres-Unda J, Zarrazquin I, Gil J, Ruiz F, Irazusta A, Kortajarena M, Seco J, Irazusta J (2013). Anthropometric, physiological and maturational characteristics in selected elite and non-elite male adolescent basketball players. J. Sports Sci..

[44] Gallahue, D. L., Ozmun, J. C. & Goodway, J. D. *Compreendendo o Desenvolvimento Motor: Bebês, Crianças, Adolesecntes e Adultos* (2013).

[45] Manuel Clemente F, Conte D, Sanches R, Moleiro CF, Gomes M, Lima R (2019). Anthropometry and fitness profile, and their relationships with technical performance and perceived effort during small-sided basketball games. Res. Sports Med..

[46] Bellows LL, Davies PL, Anderson J, Kennedy C (2013). Effectiveness of a physical activity ıntervention for head start preschoolers: A randomized ıntervention study. Am. J. Occup. Ther..

[47] Matvienko O, Ahrabi-Fard I (2010). The effects of a 4-week after-school program on motor skills and fitness of kindergarten and first-grade students. Am. J. Health Promot..

[48] Raju, S., Rengasamy, S., Sia Seng Lee, W. A., Varatharajoo, C. & Sukumaran, S. D. The effect of an after school intervention program on selected health-related physical fitness components among secondary school boys. in *Persidangan Kebangsaan Kurikulum Dan Teknologi Pengajaran* (University of Malaya, 2014).

[49] Domaradzki J, Koźlenia D, Popowczak M (2022). The mediation role of fatness in associations between cardiorespiratory fitness and blood pressure after high-intensity interval training in adolescents. Int. J. Environ. Res. Public Health.

[50] Popowczak M, Rokita A, Koźlenia D, Domaradzki J (2022). The high-intensity interval training introduced in physical education lessons decrease systole in high blood pressure adolescents. Sci. Rep..

[51] Acero RM, Olmo MF, Sánchez JA, Otero XL, Aguado X, Rodríguez FA (2011). Reliability of squat and countermovement jump tests in children 6 to 8 years of age. Pediatr. Exerc. Sci..

[52] Martín-Moya R, Silva AF, Clemente FM, González-Fernández FT (2023). Effects of combined plyometric, strength and running technique training program on change-of-direction and countermovement jump: A two-armed parallel study design on young soccer players. Gait Posture.

[53] Focke A, Strutzenberger G, Jekauc D, Worth A, Woll A, Schwameder H (2013). Effects of age, sex and activity level on counter-movement jump performance in children and adolescents. Eur. J. Sport Sci..

[54] McMahon J, Rej S, Comfort P (2017). Sex differences in countermovement jump phase characteristics. Sports.

[55] Lourenço J, Rodrigues C, Flôres F, Soares D (2022). Physical activity time and intensity in physical education during the covıd-19 pandemic. Percept. Motor Skills.

[56] Dragonea P, Zacharakis E, Kounalakis S, Kostopoulos N, Bolatoglou T, Apostolidis N (2019). Determination of the exercise intensity corresponding with maximal lactate steady state in high-level basketball players. Res. Sports Med..

[57] Rickard K, Gallahue DL, Gruen GE, Tridle M, Bewley N, Steele K (1995). The play approach to learning in the context of families and schools. J. Am. Diet. Assoc..

[58] Robinson LE, Stodden DF, Barnett LM, Lopes VP, Logan SW, Rodrigues LP, D’Hondt E (2015). Motor competence and its effect on positive developmental trajectories of health. Sports Med..

